# Authors’ Response to Peer Reviews of “Selection of the Optimal L-asparaginase II Against Acute Lymphoblastic Leukemia: An In Silico Approach”

**DOI:** 10.2196/33217

**Published:** 2021-09-08

**Authors:** Adesh Baral, Ritesh Gorkhali, Amit Basnet, Shubham Koirala, Hitesh Kumar Bhattarai

**Affiliations:** 1 Department of Biotechnology Kathmandu University Dhulikhel Nepal

*This is the authors’ response to peer-review reports for the paper “Selection of the Optimal L-asparaginase II Against Acute Lymphoblastic Leukemia: An In Silico Approach”.*

## Round 1 Review

### Authors’ Response to Anonymous [1]

#### Major Comments

1. The formatting has been changed to make the paper [2] more concise and in line with proper journal formatting standards.
2. We constructed our tree using the maximum likelihood method. Maximum likelihood is a probability-based phylogenetic tree construction method. It allows the user to choose a model of evolution and constructs the tree based on the probabilities associated with the sequences. The maximum likelihood method considers a tree more preferable if the sequences are more probable in that tree. Thus, it is a sequence-based tree.
  Lines 15-26 of page 9: We have added to the paper our reasoning and relevant literature references explaining how sequence-only–based screening is sufficient to link immunology in our study.
3. The number of initial candidates studied, numbers of candidates selected/screened, and the reasoning behind their selection has been added for each step where screening took place.
  Lines 9-14 of page 9: Screening based on tree
  Lines 5-7 of page 22: Screening from docking and binding energy

#### Minor Comments

1. Page 10: A higher quality version of the phylogenetic tree was added to the manuscript, and squares were used for highlighting.
2. Lines 7 and 8 of Page 9: The mistakes were corrected, and the positions of the commercially available (at the top) and our candidate (at the bottom) organisms mentioned in accordance with the tree (and its legend) is present in the manuscript.
3. Sites were identified by superimposing and aligning the candidate sequences with the sequence of 1nns asparaginase using PyMOL. This has been explained in the Methods section (line 11 of page 7).
4. Page 19: * was replaced with proper multiplication signs in the table.

### Authors’ Response to Reviewer S [3]

#### Major Comments

1. Lines 11-14 of page 6: An explanation of Blastp was added. The purpose of using blastp in this method is explained.
2. Lines 4-10 of page 23: A figure of the sequence alignment of our optimal enzyme candidates and the *E coli* (subject) sequence has been added with an explanation.
3. Lines 8-25 of page 29: A segment regarding relevant studies that support our findings was added to the Discussion section. The added segment uses previous studies on enzyme screening/optimization, especially L-asparaginase, to support the tools we used and the results we have obtained.
4. Line 17 of page 31: The Conclusion section was edited to be clearer on the finding of this study. A proper summery of our work and our results were added.

#### Minor Comments

1. Lines 5-7 of page 7: The full forms and meaning of DOPE and SOAP have been added.
2. Line 6 of page 30: The 6 species with Kms have been added to the Discussion section.
3. Line 26 of page 30: The sentence was rewritten to be clearer on its meaning.

### Authors’ Response to Reviewer T [4]

#### Major Comments

We have edited all our figures to remove unnecessary parts and make them appropriately compact. Several related figures have been combined together for compactness.

#### Minor Comments

1. Line 2 of page 3: A peer-reviewed paper was cited.
2. A space was added between the text and citation.
3. Lines 7-11 of page 3: The paragraph was rewritten. A better and more contextual opening sentence was used.
4. Line 26 of page 4: Analyses was changed to analyze.
5. Pages 12 and 13: Plot labelled for species; figures edited to only include relevant information.
6. Pages 14-16: Arrows added to highlight points.
7. Pages 14-16: The three figures were combined and edited.
8. Pages 25 and 26: Species’ names were added to each panel. Multiple figures were combined and edited to be more compact.
9. Page 27: All figures were combined into one. The figures were edited to be more compact.

## References

[1] Anonymous (2021). Peer review of "Selection of the Optimal L-asparaginase II Against Acute Lymphoblastic Leukemia: An In Silico Approach". JMIRx Med.

[2] Baral A, Gorkhali R, Basnet B, Koirala S, Bhattarai HS (2021). Selection of the optimal L-asparaginase II against acute lymphoblastic leukemia: an in silico approach. JMIRx Med.

[3] Hardika N (2021). Peer review of "Selection of the Optimal L-asparaginase II Against Acute Lymphoblastic Leukemia: An In Silico Approach". JMIRx Med.

[4] Mohammad A (2021). Peer review of "Selection of the Optimal L-asparaginase II Against Acute Lymphoblastic Leukemia: An In Silico Approach". JMIRx Med.

